# Administration of Jerusalem artichoke reduces the postprandial plasma glucose and glucose-dependent insulinotropic polypeptide (GIP) concentrations in humans

**DOI:** 10.29219/fnr.v66.7870

**Published:** 2022-04-04

**Authors:** Hirokazu Takahashi, Akane Nakajima, Yuichi Matsumoto, Hitoe Mori, Kanako Inoue, Hiroko Yamanouchi, Kenichi Tanaka, Yuki Tomiga, Maki Miyahara, Tomomi Yada, Yumiko Iba, Yayoi Matsuda, Keiichi Watanabe, Keizo Anzai

**Affiliations:** 1 Division of Metabolism and Endocrinology, Faculty of Medicine, Saga University, Saga, Japan; 2 Liver Center, Saga University Hospital, Faculty of Medicine, Saga University, Saga, Japan; 3 Center of Nutritional Education, Saga University Hospital, Faculty of Medicine, Saga University, Saga, Japan; 4 Center for Education and Research in Agricultural Innovation, Faculty of Agriculture, Saga University, Karatsu, Saga, Japan; 5 Department of Medicine and Bioregulatory Science, Graduate School of Medical Sciences, Kyushu University, Fukuoka, Japan

**Keywords:** Kikuimo, Topinambou, incretin, glucagon-like peptide-1, impaired glucose tolerance, diabetes mellitus

## Abstract

**Background:**

The consumption of Jerusalem artichoke has multiple beneficial effects against diabetes and obesity.

**Objective:**

The aim of this study was to determine the effect of a single administration of Jerusalem artichoke tubers on postprandial glycemia and the concentrations of incretin hormones in humans.

**Method:**

Grated Jerusalem artichoke was administered prior to a meal (Trial 1; white rice for prediabetic participants, *n* = 10). Dose-dependent effect of Jerusalem artichoke (Trial 2; white rice for prediabetic participants, *n* = 4) and effect prior to the fat-rich meal were also investigated (Trial 3; healthy participants, *n* = 5) in this pilot study. Circulating glucose, insulin, triglyceride, glucagon, active glucagon-like peptide-1 (GLP-1), and active glucose-dependent insulinotropic polypeptide (GIP) concentrations were subsequently measured in all the trials.

**Results:**

Jerusalem artichoke significantly reduced the glucose and GIP concentrations after the consumption of either meal in Trial 1 and Trial 3, whereas there were no differences in the insulin, glucagon, and active GLP-1 concentrations. Also, there was no significant difference in the triglyceride concentration after the ingestion of the fat-rich meal in Trial 3. The glucose and GIP-lowering effects were dose-dependent, and the consumption of at least 100 g of Jerusalem artichoke was required to have these effects in Trial 2.

**Conclusion:**

This study demonstrates that a single administration of Jerusalem artichoke tubers reduces postprandial glucose and active GIP concentrations in prediabetic and healthy individuals.

## Popular scientific summary

- A single administration of Jerusalem artichoke decreased the postprandial blood glucose and GIP concentrations.
- Blood insulin, glucagon, GLP-1 and triglyceride concentrations were not affected by an administration of Jerusalem artichoke.
- At least 100 g of Jerusalem artichoke was required to obtain these effects.

Type 2 diabetes is a common lifestyle-related disease and is increasing in prevalence globally (1, 2). The economic cost of diabetes is also increasing, including the cost of new and more expensive antidiabetic agents (3, 4). It is evident that lifestyle modification, including dietary changes, reduces the necessity for the use of pharmacological therapies against type 2 diabetes (5) and prevents the onset of type 2 diabetes (6–8). This suggests that dietary therapy, including the consumption of medicinal food items, may help reduce the prevalence of diabetes and the associated economic burden.

*Helianthus tuberosus L.*, also known as Jerusalem artichoke, is cultivated in North America, Northern Europe, East Asia, Australia, and New Zealand. Positive effects of the oral administration of Jerusalem artichoke on glycemic control have been reported in humans and other animals, such that it is recognized as a medicinal plant (9–11). Inulin is a major component of fructans and the dietary fibers present in Jerusalem artichoke, and the continuous administration of inulin isolated from Jerusalem artichoke tubers has been shown to improve glycemic control and reduce the obesity of diabetes patients and animal models (12–14). However, the effects of a single administration of entire Jerusalem artichoke tubers in an ordinary meal on postprandial glycemia have not been investigated.

The incretin hormones glucagon-like peptide-1 (GLP-1) and glucose-dependent insulinotropic polypeptide (GIP) regulate glucose homeostasis and are recognized as important therapeutic targets for diabetes and obesity (15, 16). After a meal, GLP-1 and GIP secreted from intestinal K- and L-cells regulate pancreatic β-cell function, but are rapidly degraded by dipeptidyl peptidase-4 (DPP-4). To date, the effect of the administration of Jerusalem artichoke on the secretion of incretin hormones has not been investigated.

In this study, we aimed to investigate the effect of the oral administration of Jerusalem artichoke tubers on postprandial glycemia and the concentrations of incretin hormones in humans.

## Materials and methods

### Participants

**Fig. 1:**
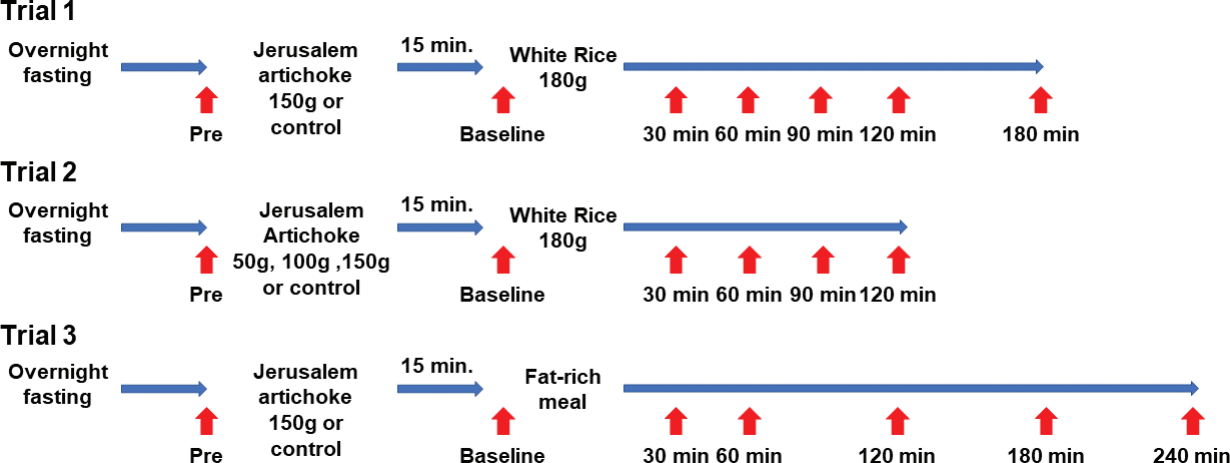
Study design.

Participants were independently recruited for three trials (Table 1 and Fig. 1). For Trials 1 and 2, prediabetic patients were enrolled. Prediabetes was defined according to the fasting plasma glucose criteria of the American Diabetes Association (17), and all patients included in Trials 1 and 2 showed fasting plasma glucose 100–125 mg/dL at least twice before inclusion and confirmed at the inclusion. For Trial 3, non-diabetic male participants with normal fasting plasma glucose concentrations < 100 mg/dL were enrolled. All the participants were recruited at Saga University Hospital between August 2016 and September 2018. The inclusion criteria for the trials were as follows: 1) age 20–80 years, 2) no existing medication with hypoglycemic and/or hypolipidemic agents, 3) no regular intake of Jerusalem artichoke within the 3 months preceding recruitment, and 4) no history of allergy to Jerusalem artichoke. The exclusion criteria for the trials were as follows: 1) known immunocompromisation or the presence of any disease or condition that might compromise participant safety, 2) pregnancy or lactation, 3) systemic treatment with corticosteroids or other immunosuppressive medication, 4) history of malignancy within the 5 years preceding recruitment, 5) history of gastrointestinal surgery, and 6) any concurrent clinically significant condition that might affect glucose or lipid metabolism. For Trial 1, the sample size was calculated according to the previous study (12), and we determined that enrollment of eight patients was needed with 90% power. We accounted for a dropout rate of up to 20%, and finally, 10 patients were enrolled. Trial 2 was conducted as does the finding study according to the results obtained in Trial 1. Trial 3 was designed as a pilot study to test the effect of a single consumption of Jerusalem artichoke on GIP kinetics after the loading of fat-rich meal in healthy male subjects. Written informed consent was obtained from all the participants. The study protocol was approved by the Certified Review Board of Saga University Hospital (2016-08-04, 2017-08-02, and 2018-07-02), and the study was conducted in accordance with the principles of the 1975 Declaration of Helsinki, revised in 2013; the CONSORT 2010 Statement; and the Japanese Clinical Trials Act. This research was funded by the Domestic Agricultural Product Consumption Expansion Project, Ministry of Agriculture, Forestry and Fisheries of Japan (grant numbers 28syokusan382, 29syokusan676-1, and 30syokusan513-1).

**Table 1:** Characteristics of the participants

Variable	Trial 1 *n* = 10	Trial 2 *n* = 4	Trial 3 *n* = 5
Age, years	65.5 (41–71)	65.5 (42–71)	38 (33–42)
Male/female	5/5	2/2	5/0
Body weight, kg	69.2 (54–89.4)	65.4 (56.8–88.2)	81 (70–116)
BMI, kg/m^2^	26.1 (22.9–31.0)	26.1 (25.3–31.0)	26.3 (22.9–34.3)
Fasting plasma glucose, mg/dL	104 (101–118)	102 (101–104)	93 (87–105)
Hemoglobin A1c, %	5.9 (5.1–6.6)	5.9 (5.5–6.3)	5.1 (5–5.5)
Total cholesterol, mg/dL	223 (192–283)	243 (217–283)	227 (11–271)
HDL cholesterol, mg/dL	59 (40–86)	52 (40–65)	61 (50–79)
LDL cholesterol, mg/dL	144 (84–205)	162 (141–205)	148 (102–188)
Triglyceride, mg/dL	114 (62–172)	124 (79–199)	113 (71–163)

Data are shown in median (range).

### Study design

Jerusalem artichokes (‘Kikuimo’ in Japanese or ‘sunflower potato’) were harvested in Saga prefecture and kept in a refrigerator at 4°C until they were used in the trials (Supplementary Fig. 1). All the participants received advice that they should not change their normal eating behavior. All the measurements were performed after an overnight fast. In Trial 1, 150 g of grated Jerusalem artichoke was consumed prior to 180 g of white rice (energy, 265 kcal; carbohydrate, 60.5 g; fat, 0 g; protein, 4.1 g) or white rice alone was consumed (control), to determine the effects of Jerusalem artichoke on glucose metabolism (Fig 1). In Trial 2, 50, 100, and 150 g of grated Jerusalem artichoke were consumed prior to 180 g of white rice, or 180 g of white rice alone was consumed (control), to determine whether the effects of Jerusalem artichoke are dose-dependent. In Trial 3, a fat-rich meal optimized for the Japanese diet (Kisei-meal: energy, 462 kcal; carbohydrate, 29.8 g; fat, 33.6 g; protein, 4.1 g; in 40 g of white toast, 30 g of butter, and 200 g of cow’s milk) (18) was used to determine the effect of Jerusalem artichoke on lipid metabolism. All the meals were consumed within 10 min. Venous blood samples were collected 30 min after the start of meal consumption. The time interval between the individual meal studies was at least 3 days.

### Physical examination and serum biochemical measurements

Venous blood samples were obtained after an overnight fast and used to measure fasting plasma glucose, total cholesterol, high-density lipoprotein-cholesterol (HDL cholesterol), low-density lipoprotein-cholesterol (LDL cholesterol), triglyceride, and insulin concentrations, and glycated hemoglobin (HbA1c) using conventional laboratory techniques. For the measurement of plasma glucagon, GLP-1, and GIP concentrations, blood samples were withdrawn directly into BP800 blood collection tubes (catalog no. 366421; BD, Minato-Ku, Tokyo, Japan) containing a DPP-4 inhibitor to prevent the degradation of GLP-1 and GIP by DPP-4, and the tubes were kept on ice until centrifugation. Glucagon was measured using an ELISA kit (catalog no. 10-1271-01; Mercodia, Winston Salem, NC, USA), according to the manufacturer’s protocol. The concentrations of total and active GLP-1 and GIP were measured using the following assay kits, as previously described (19, 20): a Human Total GLP-1 kit (catalog no. K111-FC1; Meso Scale Discovery, Gaithersburg, MD, USA), a Glucagon-Like Peptide-1 (Active) ELISA kit (catalog no. EGLP-35K; Millipore, Billerica, MA, USA), a Human Total GIP kit (catalog no. EZHGIP-54K; Millipore), and a Human GIP Active Form Assay kit (catalog no. 27201; IBL, Fujioka, Gunma, Japan). All the laboratory testing was performed at an independent central laboratory (Kyushu Pro Search LLP, Fukuoka, Japan).

### Statistical analyses

Data are presented as medians and ranges. The Wilcoxon signed-rank test and two-way analysis of variance (ANOVA) were used to compare quantitative data between the meals. Differences were considered significant when *P* < 0.05. All analyses were performed using SPSS Statistics version 21 (IBM, Inc., Armonk, NY, USA).

## Results

### Effect of Jerusalem artichoke on postprandial plasma glucose

**Fig. 2:**
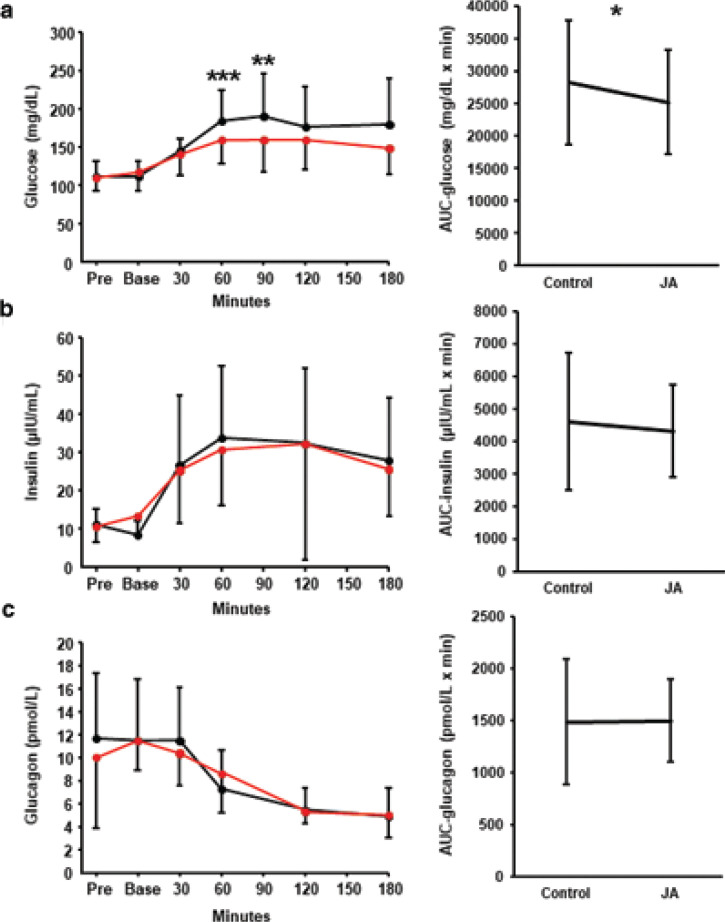
Effects of Jerusalem artichoke on glucose metabolism. Jerusalem artichoke (JA) was administered prior to a white rice meal, or the rice was consumed alone, and the subsequent plasma (a) glucose, (b) insulin, and (c) glucagon concentrations were compared. Black dot and line: control meal; red dot and line: meal preceded by JA consumption. Data are means and standard deviations. *P < 0.05, **P < 0.01, and ***P < 0.001 versus control, according to the Wilcoxon signed-rank test.

In Trial 1, the plasma glucose, insulin, and glucagon concentrations were measured after the consumption of white rice ± Jerusalem artichoke. There were no significant changes in these parameters between the baseline and postprandial measurements. Compared to the control meal, the plasma glucose was significantly lower at 60 min (184 ± 39.1 mg/dL vs. 159 ± 31.6 mg/dL, *P* < 0.001) and 90 min (190 ± 56.0 mg/dL vs. 159 ± 41.1 mg/dL, *P* = 0.004) after the consumption of the white rice meal, when Jerusalem artichoke was consumed 15 min beforehand (Fig. 2a). Two-way ANOVA confirmed the significant intervention effect and timing effect (*P* = 0.044 and *P* < 0.001 for 60 min: *P* = 0.047 and *P* < 0.001 for 90 min). The area under the curve (AUC) for plasma glucose after Jerusalem artichoke consumption was significantly smaller than that for the control meal (*P* = 0.016). However, there was no significant difference in the plasma insulin or glucagon concentrations between the test and control trials (Fig. 2b, c).

### Effect of Jerusalem artichoke on plasma incretin hormone concentrations

**Fig. 3:**
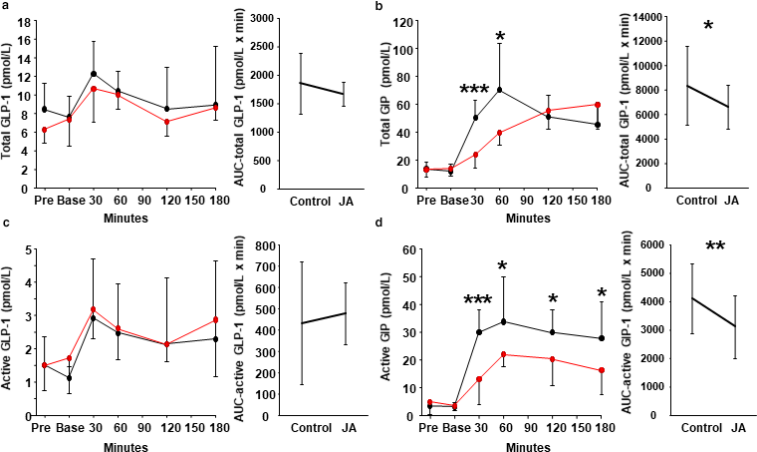
Effects of Jerusalem artichoke on incretin hormone concentrations. Jerusalem artichoke (JA) was administered prior to a white rice meal, or white rice was consumed alone, and the subsequent plasma (a) total glucagon-like peptide-1 (GLP-1), (b) total glucose-dependent insulinotropic polypeptide (GIP), (c) active GLP-1, and (d) active GIP concentrations were compared. Black dot and line: control meal; red dot and line: meal preceded by JA consumption. Data are means and standard deviations. *P < 0.05, ** *P* < 0.01, and ***P < 0.001 versus control, according to the Wilcoxon signed-rank test.

In Trial 1, the concentrations of total and active GLP-1 and GIP were also measured. There was no significant difference in total GLP-1 between the control and Jerusalem artichoke meals (Fig. 3a). However, when Jerusalem artichoke was consumed before the white rice, the total GIP concentration was significantly lower 30 min after the meal (50.1 ± 12.9 pmol/L vs. 23.5 ± 9.3 pmol/L, *P* < 0.001) (Fig. 3b), and intervention effect (*P* < 0.001) and timing effect (*P* < 0.001) were also significant at this time point in two-way ANOVA. Similarly, there was no significant difference in the active GLP-1 concentration (Fig. 3c), but the active GIP concentration was significantly lower from 30 to 180 min after the meal when Jerusalem artichoke was consumed beforehand (Fig. 3d), and intervention effect (*P* < 0.001 for 30 min, *P* = 0.006 for 90 min, *P* = 0.024 for 120 min and *P* = 0.046 for 180 min) and timing effect (*P* < 0.001 for 30, 60, 120 and 180 min) were also significant in two-way ANOVA.

### The effects of Jerusalem artichoke on glucose metabolism and GIP concentration are dose-dependent

**Fig. 4:**
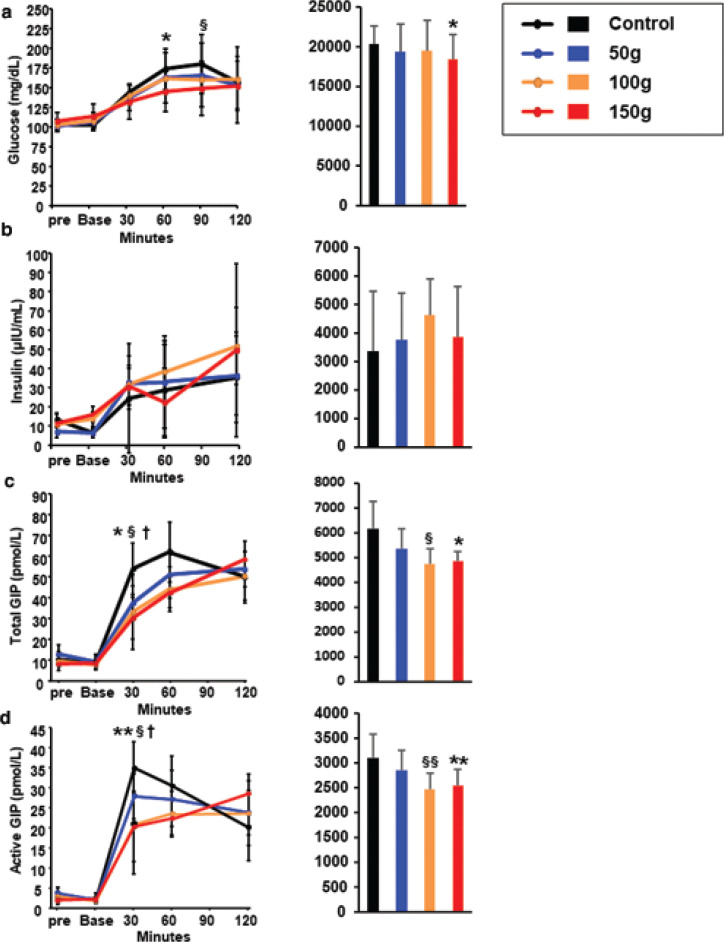
Jerusalem artichoke has dose-dependent effects on glucose metabolism and glucose-dependent insulinotropic polypeptide (GIP) concentration. Jerusalem artichoke (50, 100, and 150 g) was administered prior to a white rice meal, or white rice was consumed alone, and the plasma (a) glucose, (b) insulin, (c) total GIP, and (d) active GIP concentrations were compared. Black, blue, orange, and red dots, lines, and bars represent the control meal, 50 g Jerusalem artichoke, 100 g Jerusalem artichoke, and 150 g Jerusalem artichoke, respectively. Data are means and standard deviations. ^†^*P* < 0.05 for control versus 50 g, ^§^*P* < 0.05 for control versus 100 g, and *P < 0.05 and **P < 0.01 for control versus 150 g, according to the Wilcoxon signed-rank test.

To investigate the dose-dependency of the effects of Jerusalem artichoke, 50, 100, and 150 g of Jerusalem artichoke were consumed prior to white rice, and the plasma glucose and GIP concentrations were compared to those following the ingestion of white rice alone, as Trial 2. Similar to the results of Trial 1, 150 g of Jerusalem artichoke reduced the glucose concentration at 60 min (*P* = 0.046) and 100 g reduced it at 90 min (*P* = 0.043) versus the control meal. In addition, the AUC glucose following the consumption of 150 g Jerusalem artichoke was significantly smaller than that associated with the control meal (Fig. 4a). There were no significant effects on the plasma insulin concentration of any amount of Jerusalem artichoke (Fig. 4b). The concentrations of total and active GIP following the consumption of 100 and 150 g Jerusalem artichoke were significantly lower than those following the control meal at 30 min (Fig. 4c and d).

### Effect of Jerusalem artichoke consumed before a fat-rich meal

**Fig. 5:**
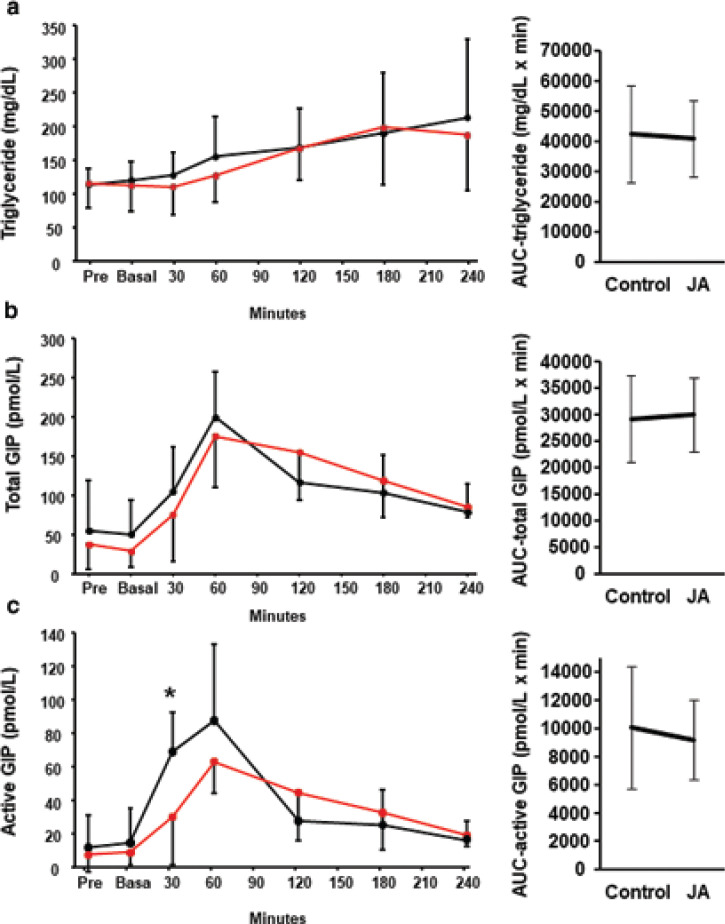
Effect of Jerusalem artichoke before the intake of fat-rich meal on the postprandial plasma triglyceride and glucose-dependent insulinotropic polypeptide (GIP) concentrations. Jerusalem artichoke (JA) was administered prior to a high-fat meal, or the high-fat meal was consumed alone, and the postprandial plasma (a) triglyceride, (b) total GIP, and (c) active GIP concentrations were compared. Solid black dot and line: control meal; red dot and line: meal preceded by the administration of JA. Data are means and standard deviations. *P < 0.05, according to the Wilcoxon signed-rank test.

The plasma triglyceride concentration was measured after the consumption of the fat-rich meal (Fig. 5a). There was no significant difference between the plasma triglyceride concentration following the control meal and the meal plus Jerusalem artichoke. In addition, there was no significant difference in the total GIP concentration. However, Jerusalem artichoke consumption significantly reduced the active GIP concentration 30 min after the start of the meal (69.0 ± 23.2 pmol/L vs. 30.1 ± 28.5 pmol/L, *P* = 0.042), and the intervention effect and timing effect were also significant at this time point (*P* = 0.029 and *P* < 0.001 in two-way ANOVA). Similar to the results of Trials 1 and 2, using white rice as the meal, Jerusalem artichoke consumption beforehand significantly reduced the plasma glucose concentration 60 min after the fat-rich meal, but it had no significant effect on the insulin concentration (Supplementary Fig. 2).

## Discussion

In this study, a single oral administration of Jerusalem artichoke significantly reduced the postprandial plasma glucose concentration in prediabetic individuals. Moreover, we have demonstrated that Jerusalem artichoke consumption reduces the postprandial plasma GIP concentration, but not that of GLP-1. These results confirm the beneficial effects of Jerusalem artichoke on glucose metabolism and demonstrate a novel effect on an incretin hormone.

The mechanism of the antidiabetic effect of Jerusalem artichoke has been investigated in humans and rodent models. The inhibition of alpha-glucosidase is considered to be a major mechanism of its effect to reduce postprandial glycemia. Alpha-glucosidase is a glucosidase enzyme that catalyzes the hydrolysis of starch to simple sugars in the intestines (21). Therefore, alpha-glucosidase inhibition reduces the rate of glucose absorption by delaying carbohydrate digestion and reduces postprandial glycemia independently of insulin (22). According to the results of previous studies, the tubers, flowers, and leaves of Jerusalem artichoke inhibit alpha-glucosidase and have an antioxidant effect (23–25), but the mechanism is not fully understood. However, these effects of Jerusalem artichoke are likely to be, at least in part, mediated by caffeoylquinic acids (26, 27). It has been reported that caffeoylquinic acid reduces alpha-glucosidase activity (26), induces the nuclear factor erythroid 2-related factor 2-antioxidant response, and attenuates oxidative stress-mediated cell death *in vitro* (28). Oxidative stress has a primary role in the pathogenesis of diabetes and diabetic complications (29). However, because the effect of antioxidants on the pathogenesis of diabetes and glycemic control is generally chronic in nature, the immediate glucose-lowering effect of a single dose of Jerusalem artichoke in the absence of a change in insulin secretion, as shown in this study, could be explained by an alpha-glucosidase inhibitory effect, rather than an antioxidant effect of the plant material.

It has been reported that the oral administration for 2 months of inulin extracted from chicory root improves the glycemic control of female patients with type 2 diabetes (14). The mechanism of this glucose-lowering effect of inulin, regardless of its source, has not been well elucidated (12, 30). Moreover, several studies have shown no glucose-lowering effect of 4 weeks’ administration of oral inulin extracted from sugar beet (31) or of the consumption for 20 days of inulin extracted from chicory root (32) in patients with type 2 diabetes. Therefore, the effects of inulin consumption on glycemia might be affected by the duration of the intervention and indirect effects, including a reduction in body weight (33) and alterations to the microbiota (34, 35). The results of a single administration have been little studied, but it has been reported that an extract of Jerusalem artichoke that included inulin reduces postprandial glycemia (12), which is consistent with the results of this study. However, Tarini et al. reported that the oral administration of 24 g inulin purified from chicory root did not have a postprandial glucose-lowering effect (36). Therefore, the postprandial glucose-lowering effect of a single administration of Jerusalem artichoke might be mediated by multiple ingredients of Jerusalem artichoke tubers or interactions between components.

The effect of the administration of Jerusalem artichoke on incretin hormone concentrations was a novel finding of this study. Jerusalem artichoke consumption before either a white rice meal or a fat-rich meal significantly reduced the plasma active GIP concentration, whereas there was no significant effect on the plasma GLP-1 concentration. Recently, an increase in GLP-1 following the ingestion of inulin-enriched soymilk has been reported in healthy adult men (37). In addition, the administration of a combination of beta-galacto-oligosaccharides and inulin to neonatal rats increased their serum GLP-1 concentration and the number of GLP-1 positive L-cells in their intestines (38). However, the administration of purified inulin failed to increase the circulating GLP-1 concentration in humans (36, 39). Taking all these findings together, it seems that the administration of inulin or Jerusalem artichoke tubers alone does not increase GLP-1 secretion, and further research is required to identify the components of plant materials that increase GLP-1 concentration in combination with inulin or Jerusalem artichoke. In contrast, Jerusalem artichoke reduced the postprandial GIP concentration in this study, as well as in a previous study in which purified inulin was administered (36). Both GLP-1 and GIP increase insulin secretion by pancreatic β-cells (15), but GIP has also been reported to be an ‘obesity hormone’. A deficiency of the GIP receptor in mice causes lower glucose uptake by adipocytes under high-fat diet-feeding conditions and resistance to high-fat diet-induced obesity (40, 41). The serum GIP concentration increases with increases in body mass index (BMI) in humans with type 2 diabetes (42), and it has been shown to increase the blood flow to adipose tissue and increase lipid deposition (43). Therefore, the lower postprandial GIP concentration induced by the consumption of Jerusalem artichoke might contribute to its anti-obesity effect.

The mechanism of the reduction in GIP induced by Jerusalem artichoke administration remains to be determined. Jerusalem artichoke consumption prior to a white rice meal reduced the concentrations of both total and active GIP, but administration prior to a fat-rich meal only reduced the active GIP concentration. These findings suggest that Jerusalem artichoke might differentially affect the secretion and degradation of GIP according to the composition of the meal: the secretion of GIP might be suppressed after a carbohydrate-rich meal, and the degradation of GIP might be promoted after a fat-rich meal, independent of any effects on GLP-1 or insulin. There are several limitations in this study. The sample size was not calculated for Trials 2 and 3, and statistical power was not adequate in these pilot studies. For Trial 3, only young male subjects without prediabetes were enrolled, and the effect of Jerusalem artichoke on the kinetics of GIP after a fat-rich meal in diabetic condition remains unclear. Further study with a larger sample size is needed to validate this study.

## Conclusion

In conclusion, this study demonstrates that a single oral administration of Jerusalem artichoke tubers reduces the postprandial glucose and active GIP concentrations in prediabetic individuals. These effects of Jerusalem artichoke may help prevent or ameliorate diabetes and obesity. Further studies are required to determine the mechanisms of these effects and to identify the active components of the tubers.

## Supplementary Material

Administration of Jerusalem artichoke reduces the postprandial plasma glucose and glucose-dependent insulinotropic polypeptide (GIP) concentrations in humans

## Supplementary materials

Supplementary Fig. 1: Picture of Jerusalem artichoke, ‘Sunflower Potato’, used in all the trials. Supplementary Fig. 2: Effect of Jerusalem artichoke on glucose metabolism in Trial 3, following the ingestion of a fat-rich meal.
