# Supplementary material for: Administration of Jerusalem artichoke reduces the postprandial plasma glucose and glucose-dependent insulinotropic polypeptide (GIP) concentrations in humans
Source: Food Nutr Res. 2022 Apr 4;66:10.29219/fnr.v66.7870. doi: 10.29219/fnr.v66.7870 (PMC8985572; doi:10.29219/fnr.v66.7870)
Supplement: Administration of Jerusalem artichoke reduces the postprandial plasma glucose and glucose-dependent insulinotropic polypeptide (GIP) concentrations in humans [file FNR-66-7870-s001.docx]

**Supplementary material**

**Supplementary Figure 1**

**
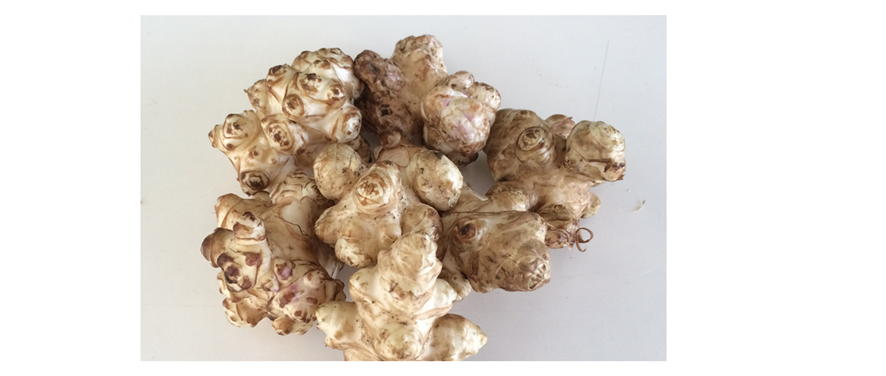
**

A strain of Jerusalem artichoke (Kikuimo in Japanese), “Sunflower Potato” was harvested in Saga prefecture and tubers were used for all the trials in the present study.

**Supplementary Figure 2**


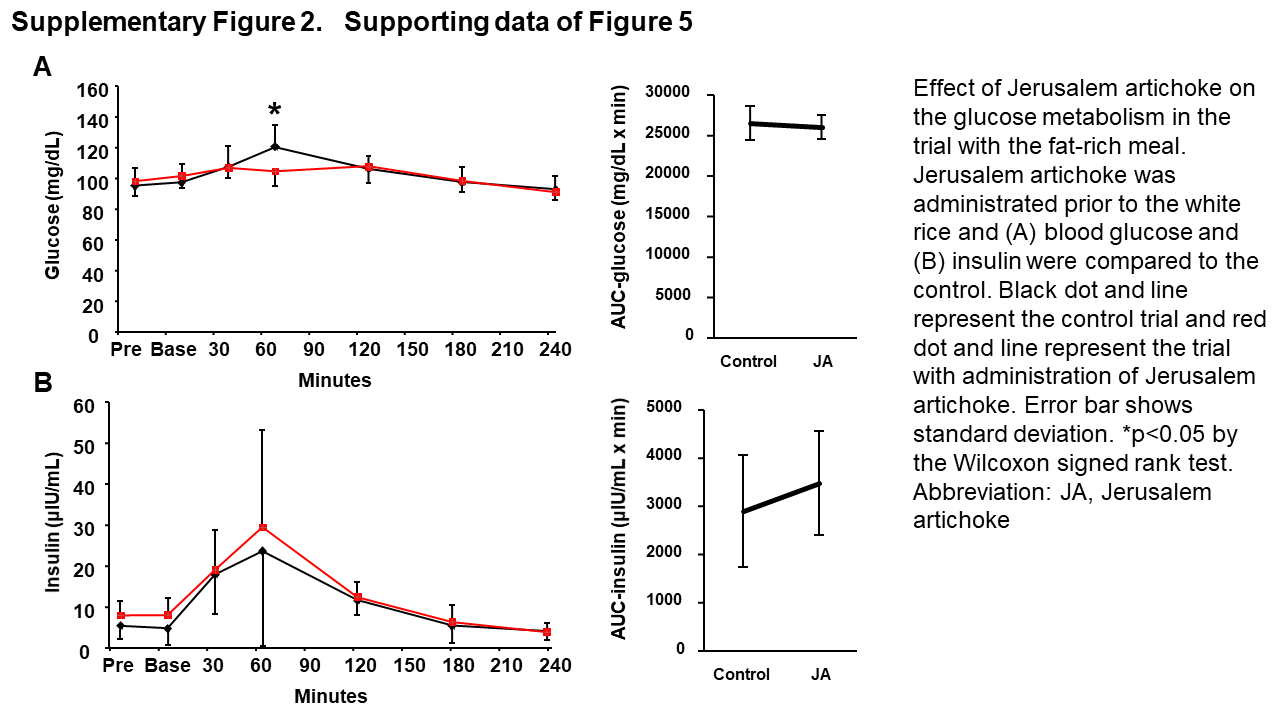


Effect of Jerusalem artichoke on glucose metabolism in Trial 3, following the ingestion of a fat-rich meal. Jerusalem artichoke was administered prior to the meal or not (control) and the subsequent plasma (A) glucose and (B) insulin concentrations were compared. Black dot and line: control meal; red dot and line: meal plus Jerusalem artichoke. Data are means and standard deviations. **p*<0.05, according to the Wilcoxon signed rank test. Abbreviation: JA, Jerusalem artichoke.
